# Fast and Efficient Screening for Wheat Loss-of-Gene Mutants Using Multiplexed Melt Curve Analyses

**DOI:** 10.1371/journal.pone.0159955

**Published:** 2016-07-26

**Authors:** Jos C. Mieog, Jean-Philippe F. Ral

**Affiliations:** CSIRO Agriculture, GPO Box 1600, Canberra, ACT, 2601, Australia; Huazhong University of Science & Technology(HUST), CHINA

## Abstract

This study describes a new approach in the screening for loss-of-gene mutants in Heavy Ion Bombardment (HIB) mutant populations of genetically complex organisms such as hexaploid bread wheat using multiplexed single-color (SYBR Green) melt curve analyses. The assay was set up for three target genes to test its validity and applicability. For each gene, three genome-specific primer pairs (one for each genome) with distinct melt curves were developed and multiplexed. This allowed screening for “single null mutants” (plants with the target gene deleted in one of the three genomes) for all three genomes in a single reaction. The first two genes (α-Amylase 3 and Epsilon Cyclase) were used to test the approach as HIB null lines for all three genomes were already available for these. The third assay was successfully applied to identify new single null lines of the target gene α-Amylase 2 in an in-house HIB wheat collection. The use of SYBR Green greatly reduced the time and/or cost investment compared to other techniques and the approach proved highly suitable for high-throughput applications.

## Introduction

The hexaploid nature of bread wheat, having 3 complete genomes, both challenges genetic research as well as provides unusual scientific opportunities. For instance, routine molecular techniques such as PCR and sequencing can be challenging as each single copy gene has 3 homeologous copies which could contain subtle single nucleotide changes, increasing the chance of undesired primer annealing and PCR anomalies (e.g. mixed sequence reads). For multi-genic families the situation is even more complex. Also, this gene redundancy can cause complications in gene knock-out strategies. On the other hand, hexaploidy makes the wheat genome highly tolerant to mutations compared to diploid plants. As a result, two types of mutant populations with high mutation frequencies have been successfully generated in wheat: 1) TILLING populations where high-frequency point mutations are randomly induced by chemical agents [1, 2], and 2) mutant populations where large deletions are created by bombardment from heavy ions (HIB for Heavy Ion Bombardment) [3]. Once identified, loss-of-function mutant lines can be introgressed and combined via a breeding program to develop double and triple loss-of-function mutants. Often, these mutants are used in studies that aim at understanding the function of the gene or the relative contribution of each of the 3 genomes to a phenotype in a classical reverse genetics approach (reviewed by Fitzgerald *et al*. [4]).

HIB mutant populations, as used in this study, are screened for lines with homozygous, genome-specific gene deletions (single null lines) in the F3 or later generations using genome-specific primer pairs. However, screening for single null lines in a bread wheat HIB population with an expected mutation rate of around 1/400, as is the case for the population used in this study [5], by conventional PCR and gel analyses can be a laborious task. Multiplexing the PCR reactions can greatly reduce the workload of setting up PCR reactions and running gels; however it also increases the chance of false positives, especially when source DNA quality and concentration varies.

A previous study developed a real-time PCR, Taqman-based approach for high-throughput detection of single null mutations in a HIB population using three separate channels [5]. Although powerful due to its sensitivity and real-time data collection, this method is relatively costly as it requires a multi-channel real-time PCR machine and uses Taqman probes. For single-copy genes it should be relatively simple to design genome-specific primers and/or probes for each genome. Therefore only a limited number of primers/probes need to be tested before the assay is set up. However, this exercise can become very challenging in the case of multi-copy genes, especially when sequence information is incomplete and/or complex as often is the case for hexaploid organisms. To overcome this challenge, suitable primer pairs have to be obtained by trialing multiple primer pairs across the gene sequence alignments which can become costly. In addition for Taqman assays, three appropriate binding sites (both primers and probe) are required per genome, potentially limiting the number of suitable target sites.

We have developed a new approach in screening for wheat null mutants using single-channel real-time PCR and multiplexed melt curve analyses. The analyses contain a check for DNA concentration/quality and produce unequivocal results for null mutants. As proof of concept, the assay was initially set up and tested to screen for deletions of a gene involved in starch degradation, α-Amylase 3 (TaAMY3) [6], and a gene involved in the carotenoid pathway, Epsilon Cyclase (TaEC) [7,8]. The rationale behind the gene selection was that for both genes the full genome-specific sequence information and HIB single (and for TaAMY3 also double) null lines were already available in our research group. Next, an assay was set up for α-Amylase 2 (TaAMY2) [9], for which neither the full sequence information nor any null lines were available, and the HIB mutant wheat population described by Li *et al*. [3] was used to screen for single null mutants for this gene.

## Materials & Methods

DNA from all combinations of the nullisomic-tetrasomic lines (in which a chromosome from a genome is replaced by an additional copy of the same chromosome of one of the two remaining genomes [10]), as well as DNA samples from wheat HIB null mutants for selected genes, were available in the lab at the beginning of the project (20-400ng/μL concentration range). Available DNA samples from wild-type Chara (~50ng/μL) were used for sequencing.

Target genes were amplified for sequencing where necessary, using genome non-specific primers to amplify the gene from all three genomes in the same reaction, using PrimeSTAR Max DNA polymerase (TaKaRa, Clonetech laboratories, Inc) according to the manufacturer’s instructions. Products were TOPO cloned using a Zero Blunt Topo PCR cloning kit (Invitrogen). Up to 14 plasmids per target gene were sequenced (BigDye Terminator v3.1 Cycle Sequencing Kit, Thermo Fisher Scientific) with the M13 forward and reverse primers to ensure good reads for all three genomes were obtained.

For TaEC, full genomic sequences for all three genomes were available in GenBank (Accession # EU649785-649787). DNA samples for single HIB null lines of each genome were also available for this gene (unpublished results).

The full TaAMY3 genome A genomic sequence was available in GenBank (Accession # X05809). Full genomic sequences of TaAMY3 for genomes B and D were amplified using the genome non-specific primers OexpAamy_for1 (TCATAGGAAGTAGAGGCGA) and OexpAamy_rev (TCAGAGGCCGCTCTTCTC) and have been submitted to Genbank (Accession # KX449312-449313). Additionally, DNA samples of both single and double HIB null lines were available for this target gene (unpublished results).

TaAMY2 was amplified using genome non-specific primers Amy2cdsF4 (GGCCACCAAGTCCTCTTTCA) and Amy2cdsR2 (CCGATCTTCACCACCACCTT). The targeted A, B and D genomic sequences have been submitted to GenBank (Accession # KX449314-KX449316). No HIB null lines were available for this target gene.

All melt runs were run on a PikoReal real-time PCR machine (Thermo Fisher Scientific) and consisted of 5 μL Sensimix SYBR no-ROX (Bioline Pty Ltd), 0.3 μL primer premix, and 3.7 μL H_2_O and 1 μL DNA template. Cycling conditions were standardized at 10 minutes @ 95°C, then 40 cycles of 10 seconds @ 95°C, 10 seconds @ 60°C, 10 seconds @ 72°C, followed by a melt curve analysis starting at 60°C and increasing at 0.2°C increments each second. Initial genome-specific test runs used forward and reverse primers premixes, both at 10nM concentrations. Multiplexed primer premixes were optimized using trial-and-error with the highest primer pair concentration pre-set at 10 nM per primer.

## Results & Discussion

### Designing melt assay primers

Using sequence information from GenBank as well as in-house generated sequences, alignments containing representative sequences for all three genomes were made for all three target genes. For the single-copy genes TaAMY3 and TaEC, the alignments contained the full gDNA sequences for all three genomes. For TaAMY2, which is a multi-copy gene, a partially resolved alignment showed at least 5 groups present in the alignment containing 31 (mostly partial) sequences.

**Fig 1:**
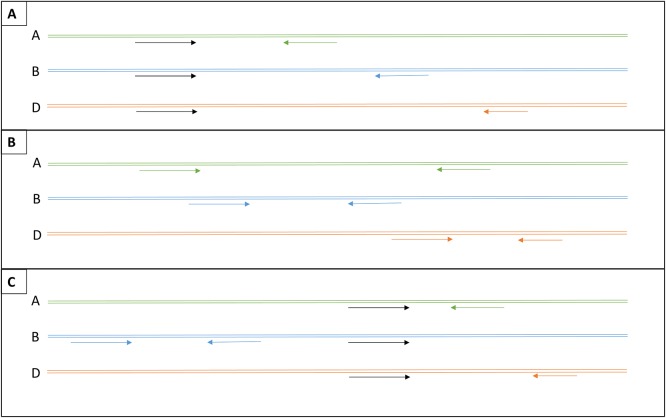
Three options for designing genome-specific primers for multiplexed melt screens. Option A shows the option with the least number of primers necessary: a genome non-specific (black) forward primer is combined with three genome-specific reverse primers (green: genome A specific, blue: genome B specific, red: genome D specific). Option B shows the option with six genome-specific primers. The last option (C) shows a possible combination of options A and B.

Genome-specific primers were designed for each genome of all three target genes (all 5 groups of the TaAMY2 alignment were targeted) according to Mieog *et al*. [11] with at least a single genome-specific SNP at the 3’ primer end. However, the aim to multiplex primer pairs with different product Tm’s required additional considerations to be taken into account. Fig 1 shows three different options (A-C) for successful multiplexing of genome-specific primer pairs. If the sequence differences between genomes allow it, multiplex option A is the preferred option because it requires the least number of primers to be multiplexed, thus reducing the likelihood of unwanted interactions between the primers. Sequence homology between the genomes generally requires different product lengths to be amplified to obtain sufficient differences between Tm’s with this approach, which may work well at intron-exon junctions. However, not all primer pairs tested may turn out to have compatible Tm’s or be genome-specific when tested using the relevant nullisomic-tetrasomic lines. The latter is especially the case when targeting multi-copy genes such as TaAMY2 with more intra-genome variability and/or when only incomplete sequence data is available. Therefore, our approach was to design primer pairs from both options A and B, with an initial testing of about 12 primer pairs (4 per genome) with a range of product Tm’s, and to add primers when necessary. Primers from options A and B can be multiplexed where required as shown in option C. However, care has to be taken that the genome non-specific primer(s) point(s) away from the other genome-specific primer pair(s) to avoid unwanted products.

Product Tm’s were estimated using the online Oligo Calc tool (http://biotools.nubic.northwestern.edu/OligoCalc.html) of which the dsDNA nearest neighbor estimates were found to be sufficiently accurate (average difference over 9 primer pairs: -0.16 ± 0.94°C).

### Testing the melt assay primers

Between twenty and thirty primers (both genome-specific and non-specific) were tested per gene in different combinations covering all three genomes. Per gene, three pairs were selected as being genome-specific (one for each genome), having compatible product melting temperatures (at least 1.5°C apart) and being suitable for multiplexing (Table 1). The first multiplex mix contained equal concentrations of each primer pair (any genome non-specific primers were counted double or triple depending on the number of reactions they were involved in).

**Table 1:** PCR primers and products details for multiplexing.

Gene	Genome	forward primer	FP_sequence	reverse primer	RP_sequence	Length (bp)	Tm (melt)
TaAMY3	A	TaAMY3_A_F	GTTCTCCAGGAGGCCGTC	TaAMY3_A_R	TCCGAGGGGAATGGCCACAG	155	87.3
TaAMY3	B	TaAMY3_B_F	GAACTGGGTGCATGGCGTCA	TaAMY3_B_R	GTCGATGAACGTGACGGTTC	166	89.1
TaAMY3	D	TaAMY3_D_F	CACATCAAATGTGCCCCATGA	TaAMY3_D_R	CGGCTTTGAGGATATCCAGTT	208	82.7
TaEC	A	TaEC_ABD_F	AGCATGGGGAACACTATGGC	TaEC_A_R	AAAGGTTTCAAAGAATGTTTGG	118	79.2
TaEC	B	TaEC_B_F	TCTCCAGATGGACATCGAGTG	TAEC_B_R	TTGAACTGGTGCAGAAACAACCT	280	83.3
TaEC	D	TaEC_ABD_F	AGCATGGGGAACACTATGGC	TaEC_D_R	CGCGAAAAAGGAAATACTTGG	204	81.6
TaAMY2	A	TaAMY2_A_F	GGGGTGGAGAACATTCTGGT	TaAMY2_ABD_R	CGCGTTGCCGTACTTGGA	222	83.5
TaAMY2	B	TaAMY_B_F	CCGGAGATGGCCAAGGTC	TaAMY_B_R	GCGGCGTTCAGAATCCCT	212	89.2
TaAMY2	D	TaAMY2_D_F	GACAAGGTCATGCAGGGCTA	TaAMY2_D_R	ACAGTGACAGCTGAGTTTCTCA	104	81.4

FP and RP are forward and reverse primer, respectively. Length refers to the PCR product in base pairs. Tm’s shown are the values obtained from the melt assays, not the predicted values.

All multiplexes were first tested on the nullosomic-tetrasomic lines as well as a Chara wild-type sample to select promising mixtures based on genome-specificity and multiplex compatibility. Generally, two peaks were visible in wild type samples and/or the different nullisomic-tetrasomic samples in this early test of successful multiplex combinations, with a third, expected in the wild-type sample, often not visible or only barely visible. Optimization steps were performed on promising mixtures with the aim to have all three melt peak heights of the Chara wild-type sample within 50% of each other. Runs with stepwise 2-fold increases/decreases (up to 8-fold) of primers whose products were too low/dominant were then undertaken, followed by more precise calibration if necessary to reach the optimal primer ratios. To minimize possible interference from non-specific peaks in the analysis, the melt temperature range for analyses was limited to the beginning of the melt peak with the lowest Tm to the end of the melt peak with the highest Tm. Note that the nullosomic-tetrasomic samples were not as good for optimization purposes due to the differences in genome-specific chromosome numbers between samples (either 0, 2 or 4 copies of the relevant chromosome per genome). For this reason, examples of melt curves shown are all from HIB DNA samples as they accurately reflect screening results.

For TaAMY3, six genome-specific primers were multiplexed (option shown in Fig 1B). The A and B specific primers annealed in exon 2, while the D specific forward primer annealed in intron 2 and the D specific reverse primer in exon 3. The optimal primer ratio was found to be 4:2:9 for genome-specific primer pairs A, B and D. For TaEC, a genome non-specific forward primer annealing in exon 7 was combined with a genome A specific reverse primer annealing in exon 7 and a genome D specific reverse primer annealing in intron 7. For genome B, two specific primers were used (forward primer in exon 3, reverse primer in intron 4) upstream from the A and D specific reactions (option shown in Fig 1C). The optimal primer ratio was found to be 12:1.5:5 for genome specific primer pairs A, B and D. For TaAMY2, an A genome specific forward primer located in intron 2 was paired with a non-specific reverse primer which annealed just after intron 2. For the D genome, the reverse primer was specific in intron 3 while the forward primer was non-specific and located just before that intron. The B primers were both genome-specific and annealed between introns 2 and 3 (option shown in Fig 1C). The optimal primer ratio was found to be 4:1:5 for genome specific primer pairs A, B and D.

**Fig 2:**
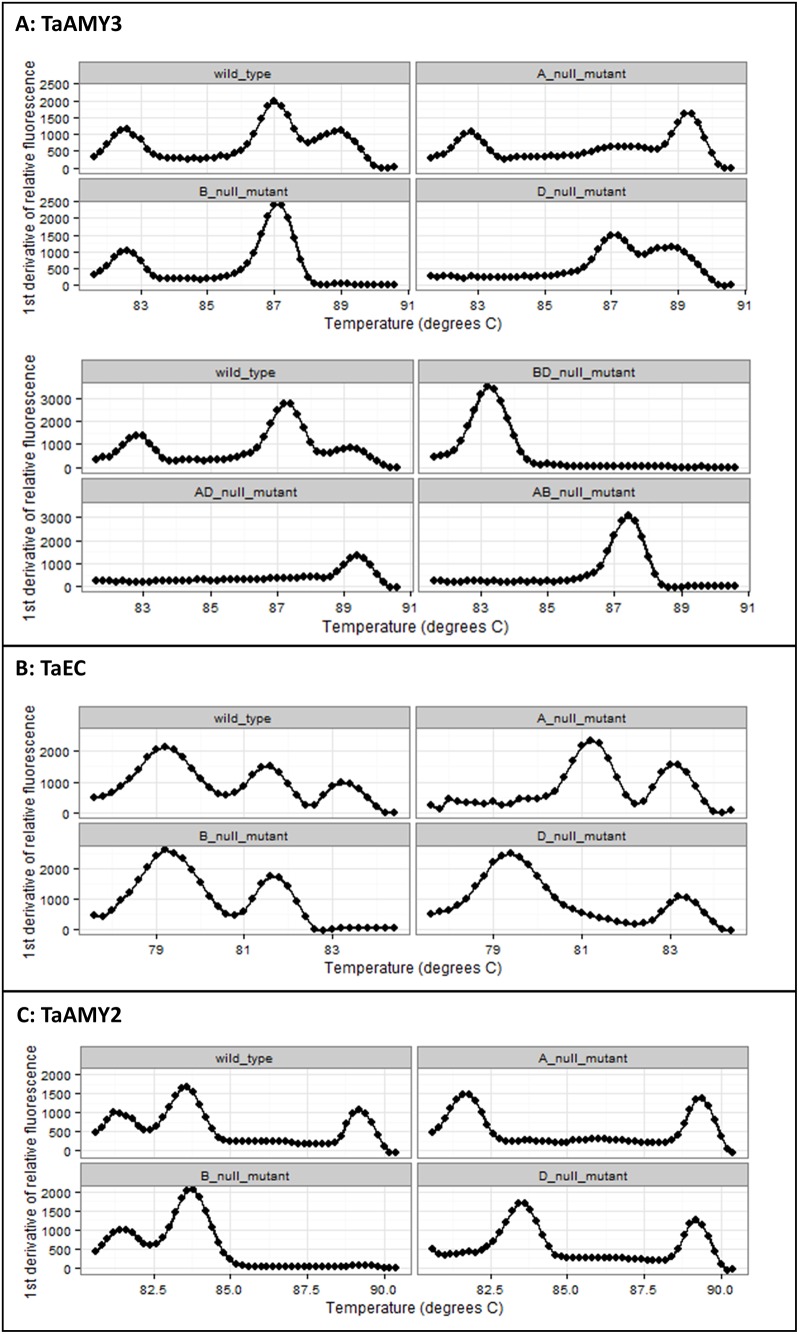
Melt curve screening results for the three target genes. Results show typical melt curves for wild type and single null mutants for the A, B and D genomes for each gene. For TaAMY3, results are also shown for double null mutants (BD_null, AD_null and AB_null).

In order to confirm that the method could be applied on a small deletion and mutagenesis population, the TaAMY3 and TaEC assays were tested on HIB wild type and single null lines from all three genomes. The TaAMY3 assay was also tested on double null lines. Fig 2A and 2B show the typical results of these screens. Results confirmed each of the genome specific mutants previously detected by conventional PCR screening and additionally confirmed the success of our breeding program for TaAMY3 identifying double mutants within our population.

### Screening the HIB mutant collection

Having successfully set up multiplexed melt assay for all three target genes, the next step was to screen the HIB population for TaAMY2 single null lines. In this assay, the wild type showed the three peaks corresponding to the genome A, B and D at melt temperatures between 83.2–83.9°C, 88.9–89.6°C and 81.0–81.7°C, respectively. For each sample separately, the PicoReal Software 2.2 designated the height and melt temperature of the highest peak as H1 and T1, the middle peak H2 and T2, and the lowest peak as H3 and T3, independent from which genome the peak represented.

During the screening process, it was found that the Ct values, which are directly linked to the DNA concentrations of the DNA samples, varied widely. Importantly, it was observed that the range of Ct values suitable for this assay was limited because the B genome specific primer pair produced higher B peaks with high DNA concentrations and lower peaks with low DNA concentrations relative to the other genome-specific peaks. As a result, samples with Ct values above 26.5 were not producing reliable results. The lowest Ct value obtained in the assay was 21.5.

To avoid having to standardize each DNA sample of the HIB population and visually inspect the result, a method was developed to analyze the data using Microsoft Excel (Table 2). With the threshold set at 250 RFU, the Ct and melt data of each sample was copied into an Excel spreadsheet. Using the filter function, samples with Ct values outside of the dynamic range (Ct > 26.5) were removed. Next, data was sorted by the smallest to largest H3 value. An unusually low H3 value relative to the other samples in the assay was indicative of a potential single null for the genome not represented by the T1 or T2 peaks. Null results were verified by visual inspection of the melt curves. Representative results for a wild type, A null, B null and D null HIB sample for TaAMY2 are shown in Fig 2C. The HIB collection was screened quickly (processing up to six 96-well plates per day) and at low cost (AU$25/plate). TaAMY2 single null mutants were detected for all genomes at an average frequency of 1/(394 ± 217).

**Table 2:** Example of a filtered Microsoft Excel spreadsheet for TaAMY2 single null mutant detection (selected data).

Sample	Plate	Well	Ct	T1	H1	T2	H2	T3	H3	Call
2	BW20A_2	A03	25.14	83.7	3780.1	81.5	1576.9	80.8	180.2	B null mutant
3	BW20A_2	A05	25.63	83.5	3071.6	89.2	2769.3	86.5	461.5	D null mutant
1	BW20A_2	A01	25.7	89.3	3242.3	81.7	2609.6	86.2	524.6	A null mutant
6	BW20A_2	A11	24.13	89.3	2715.3	83.6	2765.1	81.4	1287.9	
5	BW20A_2	A09	25.91	83.6	3321.4	81.5	1721.8	89.3	1424.4	
4	BW20A_2	A07	24.71	83.5	2956.7	89.2	2265.6	81.4	1474.1	

Reactions highlighted in green show the detected single null mutants, easily identified by their unusually low H3 value. T1 and T2 show which genome has been detected in the single null samples. For sample 1, A (= 83.9°C) and D (= 81.6°C); for sample 2, B (= 89.2°C) and D; and for sample 3, B and A. Third peaks detected in single null mutants are noise.

Data analysis is assisted by optimizing the assay so that the melt peaks for the three genomes in wild type samples are similar (ideally with peak heights within 50% of each other) within the dynamic range. As observed in the TaAMY2 assay DNA concentration may have an effect on the melt peak similarity, and thus the H3 diagnostic value, possibly due to genome-specific primer pairs having different efficiencies. In these cases, the dynamic range may be influenced by the relative primer concentrations in the multiplex and may therefore need to be optimized for specific sample collections depending on the average DNA concentration. These aspects will be different for each assay and need to be taken into consideration during the optimization process.

As TaAMY2 is a multi-copy gene [9], the melt assay as presented here only screens for null mutants for those copies of the TaAMY2 gene that are directly targeted. If the copies of the gene are adjacent in the genome, then null lines for all copies of that genome can be obtained as the HIB process induces large deletions often spanning multiple genes. However, it is possible nulls for one copy but not for another in a specific genome are detected. This can be verified by testing the lines with genome-specific primer pairs for the different copies as found in the alignment, although extensive sequencing may be necessary to full resolve the gene alignment to allow effective intra-genome primer design. Alternatively, whole-genome analyses such as 90k SNPChip analyses, available for wheat [12], may be used to verify the loss of the gene per genome in identified single null mutants. These considerations do not apply to single-copy genes.

### Other applications

Our application has been focused on detecting genome-specific null lines for a gene in hexaploid wheat in a HIB DNA sample collection. The results of the TaAMY3 assay demonstrate that the assay can be similarly beneficial in the selection of single and double nulls in F2 (or later) generations after (back)crosses. Triple nulls can be detected using genome-non-specific primers for the target gene. Also, the assay should work equally well when multiplexing for different gene deletions, for instance in diploid organisms. In these cases, it should be possible to multiplex 4 or even 5 targets within the range of Tm’s available for real-time PCR analyses (~75 to ~90°C), although obtaining suitable primers and optimizing the primer pair ratios would get progressively more complicated.

The genome-specific assay can also be used to screen PCR products and/or plasmids from polyploid organisms. This is useful when trying to obtain additional (full) target gene sequences for all genomes. The assay can test whether a putative genome non-specific primer pair amplifies all three genomes before cloning. Similarly, plasmids can be screened to determine from which genome the gene has been cloned. This way it can be assured that enough sequence information is obtained from all three genomes with minimal sequencing.

## Conclusions

The assay method presented here greatly improves the screening process for single null mutants in wheat HIB populations compared to conventional PCR and post-PCR gel-based screening analyses, and promises similar benefits in screening for single or double nulls after (back)crosses. Additionally, it has cost-benefits compared to Taqman assays in both consumables and hardware requirements. Having set up the assay for three genes without unexpected difficulties and having tested one on both single and double nulls, it promises to be widely applicable and, being optimized for cost and effort efficiency, highly suited to high-throughput applications.
